# Characterization of a novel Nav1.5 channel mutation, A551T, associated with Brugada syndrome

**DOI:** 10.1186/1423-0127-16-76

**Published:** 2009-08-25

**Authors:** Kun-Chi Chiang, Ling-Ping Lai, Ru-Chi Shieh

**Affiliations:** 1 Institute of Biomedical Sciences, Academia Sinica, Taipei, Taiwan, Republic of China; 2 Department of Internal Medicine, National Taiwan University Hospital, Taipei, Taiwan, Republic of China

## Abstract

Brugada syndrome is a life-threatening, inherited arrhythmia disorder associated with autosomal dominant mutations in SCN5A, the gene encoding the human cardiac Na^+ ^channel α subunit (Nav1.5). Here, we characterized the biophysical properties of a novel Brugada syndrome-associated Nav1.5 mutation, A551T, identified in a proband who was successfully resuscitated from an episode of ventricular fibrillation with sudden collapse. Whole-cell currents through wild-type (WT) Nav1.5 and mutant (A551T) channels were recorded and compared in the human embryonic kidney cell line HEK293T transfected with SCN5A cDNA and SCN1B cDNA, using the patch-clamp technique. Current density was decreased in the A551T mutant compared to the WT. In addition, the A551T mutation reduced Nav1.5 activity by promoting entry of the channel into fast inactivation from the closed state, thereby shifting the steady-state inactivation curve by -5 mV. Furthermore, when evaluated at -90 mV, the resting membrane potential, but not at the conventionally used -120 mV, both the percentage, and rate, of channel recovery from inactivation were reduced in the mutant. These results suggest that the DI-DII linker may be involved in the stability of inactivation gating process. This study supports the notion that a reduction in Nav1.5 channel function is involved in the pathogenesis of Brugada syndrome. The structural-functional study of the Nav1.5 channel advances our understanding of its pathophysiolgocial function.

## Background

Brugada syndrome is a life-threatening, inherited arrhythmia disorder associated with autosomal dominant mutations in SCN5A [1-4], the gene encoding the human cardiac Na^+ ^channel α subunit (Nav1.5) [5], which contains four homologous domains, each composed of six membrane-spanning segments, linked by cytoplasmic linkers. Brugada syndrome is characterized by a distinctive ST-segment elevation in the V1–V3 leads of the ECG that reflects abnormal electrical forces in the right ventricle [6], which are linked to SCN5A mutations causing reduced Nav1.5 function [5]. The discovery of SCN5A mutations in families with Brugada syndrome was first reported in 1998 [1]. Subsequently, several others have been identified and functional studies on these mutations have been performed using a heterologous expression system [2,7-11]. Despite many studies, the molecular and cellular mechanisms underlying Brugada syndrome are not completely known [12,13].

Nav1.5 channels initiate action potentials in most cardiac myocytes and thus play a critical role in cardiac excitability and impulse propagation. Some Brugada syndrome-related SCN5A mutations produce lose-of-function defects by completely disrupting Nav1.5 function [1] or by reducing ion permeation or membrane surface expression [14], whereas others elicit a functional deficit by accelerating the rates of fast and slow inactivation [7,9,14,15]. The identification of the various clinical phenotypes resulting from SCN5A mutations is critical for optimal patient management. In addition, an understanding of the structural-functional relationship of the Nav1.5 channel may result in the development of new therapies for heart diseases.

In this study, we describe the functional properties of an Nav1.5 mutation, A551T, identified in a patient with Brudaga syndrome, whose resting ECG showed a coved-type ST elevation in the right precordial leads [16]. We found that the A551T mutation decreased the Na^+ ^current density, enhanced entry into fast inactivation from the closed state, and decreased channel recovery from inactivation. The decreased Nav1.5 activity caused by the A551T mutation supports the hypothesis that a reduction in Nav1.5 function is involved in the pathogenesis of Brugada syndrome.

## Materials and methods

### Genetic analysis

Genomic DNA was purified from peripheral blood lymphocytes using Gentra Blood DNA isolation kit (Gentra, USA) after obtaining informed consent from the patient. All exons of SCN5A were amplified by polymerase chain reaction (PCR) and screened for mutations using the dideoxynucleotide chain termination method with fluorescent dideoxynucleotides on an ABI DNA sequencer (PE Applied-Biosystem, USA). The investigation conforms to the principles outlined in the Declaration of Helsinki. This study was approved by the ethics review board of the National Taiwan University Hospital (NTUH 9400000202).

### Cell culture

HEK293T cells are the Human Embryonic Kidney 293 cells, transformed by expression of the large T antigen from SV40 virus that inactivates pRb. These cells were cultured in Dulbecco's modified Eagle's medium (Sigma Chemical, St. Lois, MO, USA) containing 10% fetal bovine serum (Life Technologies, Paisley, Scotland) and 1% penicillin-streptomycin at 37°C in a humidified atmosphere containing 5% CO_2_. Cells were plated on poly-L-lysine-coated No. 1 glass cover slips (42 mm) (Carl Zeiss, Inc., Germany) and transiently transfected with SCN5A-CFP (0.75 μg) and SCN1B-YFP (0.75 μg) using LipofectAMINE 2000 (Invitrogen Co., Carlsbad, CA, USA). Cells expressing both proteins, identified by double fluorescence, were selected for experiments.

### Electrophysiological recordings

Whole-cell currents were recorded at room temperature (21–24°C) using the patch-clamp technique [17,18] and an Axopatch 200B amplifier (Axon Instruments, Foster City, CA, USA). The extracellular solution (pH 7.4, titrated with NaOH) contained (in mM): NaCl 140, CsCl 10, CaCl_2 _2, MgCl_2 _1, glucose 5, and HEPES 10. The intracellular solution (pH 7.2, titrated with CsOH) contained (in mM): CsF 110, CsCl 10, NaF 10, EGTA 11, CaCl_2 _1, MgCl_2 _1, Na_2_ATP 2, and HEPES 10. The command voltage pulses were controlled and data acquired using pClamp6 software (Axon Instruments, Foster City, CA, USA). Series resistance was compensated by ≈ 80%. The membrane potential was not corrected for liquid junction potential (-6.7 mV) because the junction potential was cancelled when calculating V_m _differences between WT and mutants. The plots of voltage dependent steady state activation and inactivation were fitted by Boltzmann equation:(1)

where V_0.5 _is the voltage at which sodium current is half-maximally activated, and k was the slope factor. Time constants of inactivation were obtained by fitting the decaying phase of current trace with a biexponential equation:(2)

The recovery from inactivation at -120 mV was fit by a biexponential equation:(3)

The recovery from inactivation at -90 mV was fit by a mono exponential equation:(4)

The voltage-dependence of all activation and inactivation recordings was recorded 5 minutes after establishing the whole-cell configuration. Data were sampled at 20 kHz and filtered at 5 kHz with the exception of those obtained during the P1 pulse, which were sampled at 6.67 kHz.

### Site-directed mutagenesis

Mutations were constructed using a PCR-based technique using overlapping primers containing the desired altered sequences. The G at base#1651 of the SCN5A gene was changed to A in the A551T mutant; the C at base#1652 of the SCN5A gene was changed to T in the A551V mutant; the C at base#1652 of the SCN5A gene was changed to A in the A551E mutant. The mutated cDNAs were sequenced using an ABI Prism™ dRhodamine Terminator Cycle Sequencing Ready Reaction Kit (PE Applied Biosystems, Foster City, CA, USA) to confirm the mutation.

### Sequence-based identification of phosphorylation sites

Potential phosphorylation sites were predicted using the NetPhos 2.0 server at http://www.cbs.dtu.dk/services/NetPhos/.

### Data analysis

Results are presented as the mean ± SEM with sample sizes (n) indicating the number of cells from which the data were obtained Statistical significance was assessed using Student's *t *test. A p value less than 0.05 was considered statistically significant.

## Results

### Clinical studies

**Figure 1:**
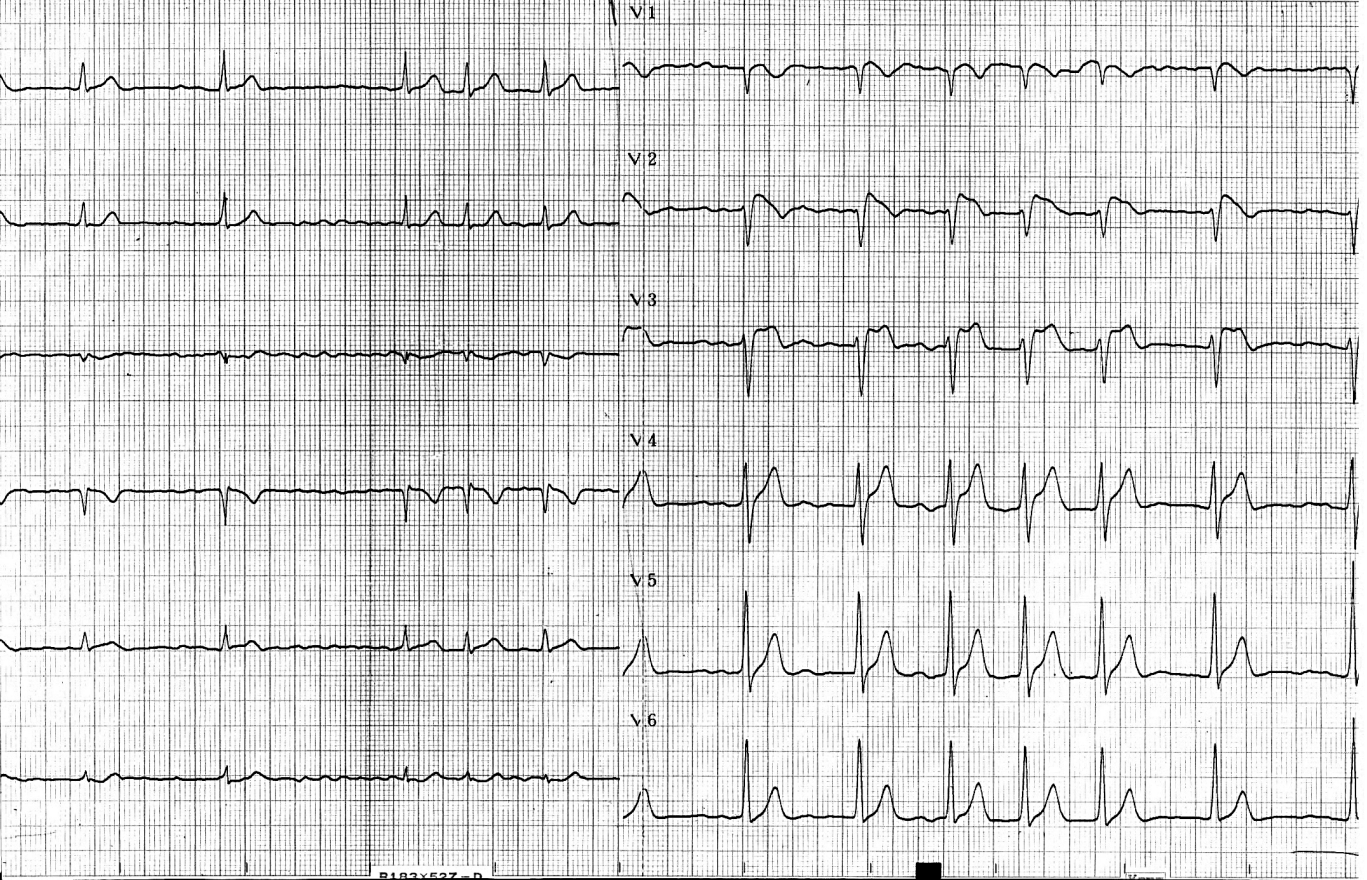
**Twelve-lead ECG of the patient with Brugada syndrome**.

The proband, a 45-year-old male patient, was successfully resuscitated from an episode of ventricular fibrillation with sudden collapse. After the episode, a 12-lead ECG was performed without a drug challenge test and showed ST segment elevation in the right precordial leads (Fig 1). In V2, the QRS complex showed a typical Brugada type 1 pattern with a coved-shape ST-T segment, while a saddleback ST-T pattern (Brugada type 2 pattern) was observed in V3. An implantable cardioverter defibrillator was implanted to prevent further attacks. Sequence analysis showed a heterozygous missense single nucleotide replacement 1651G>A, which resulted in an amino acid change from alanine to threonine at position 551 (abbreviated as A551T) in the DI-DII linker of the Nav1.5 channel [16].

### Electrophysiological characterization of the wild-type and A551T Nav1.5 channels

**Figure 2:**
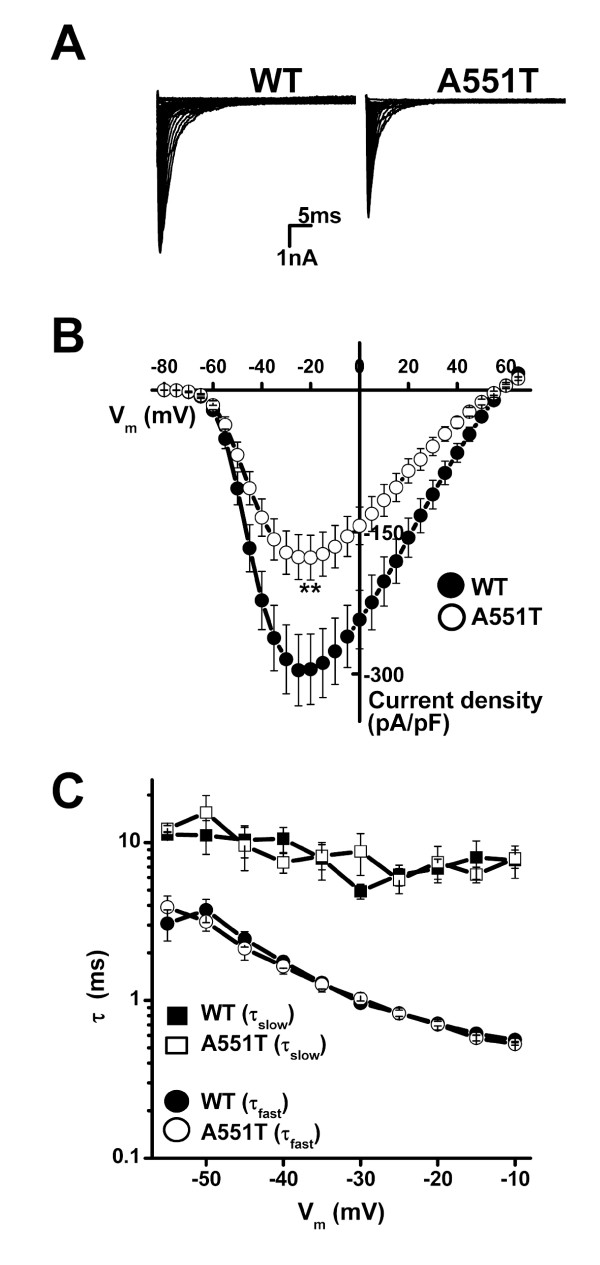
**Current density and inactivation kinetics of WT and A551T channels.  **A. Current traces recorded at Vm in the range -80 to +65 mV from a holding potential of -120 mV.  B. Averaged current density-Vm relationships.  C. Vm dependence of the inactivation time constants.  n = 18 for WT and 17 for A551T.  * p < 0.05; ** p < 0.01; *** p <0.005 vs. WT in this study.

Next, we compared the electrophysiological properties of WT and A551T channels heterologously expressed in HEK293T cells. Figure 2A shows current traces recorded at V_m _in the range -80 to +65 mV from a holding potential of -120 mV. Figure 2B shows the V_m _dependence of the averaged current density. The A551T mutation did not change the shape of I-V curve, but significantly reduced the peak current density (p < 0.01). To compare the inactivation kinetics of the WT and A551T mutant, whole-cell currents at various potentials were fitted to a bi-exponential function. Figure 2C shows that both the fast (τ_f_, 0.5 – 4 ms) and slow (τ_s_, ≈ 10 ms) inactivation time constants were the same in the WT and A551T mutant, showing that the A551T mutation did not affect the inactivation kinetics.

**Figure 3:**
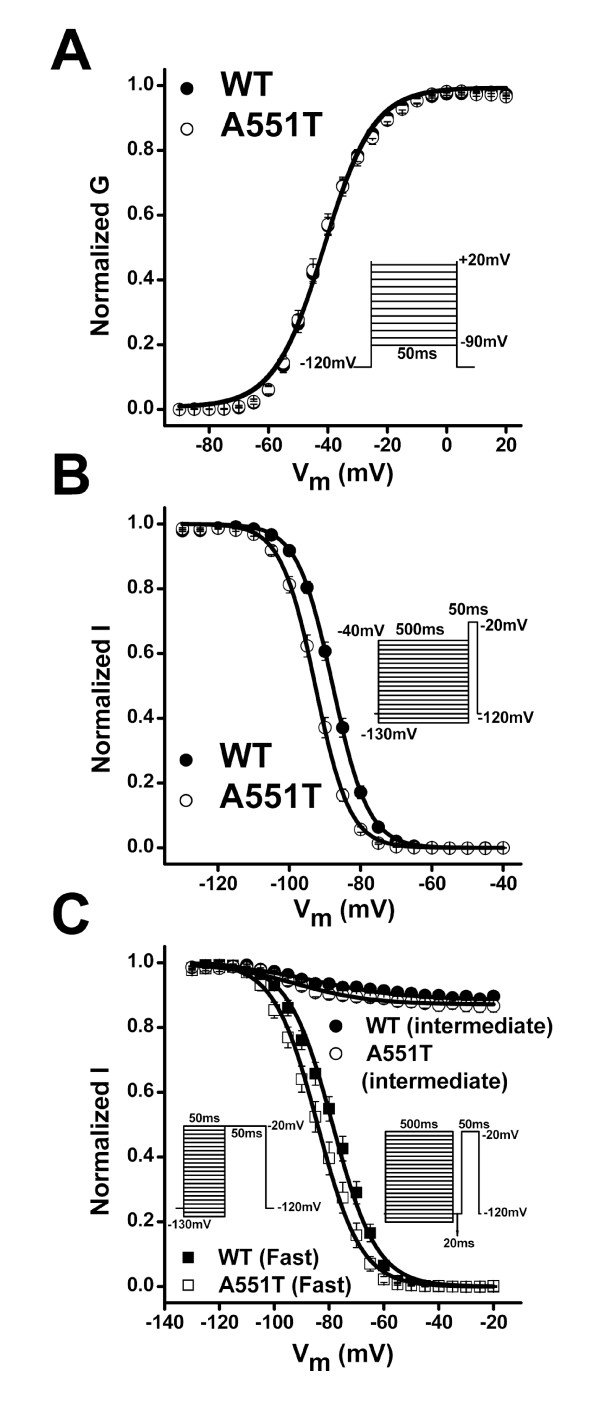
**V_m_-dependent steady-state activation and inactivation of WT and A551T channels** A. The normalized peak conductance-V_m _relationship. n = 22 for WT and 30 for A551T. B. V_m _dependence of steady-state inactivation. Currents were normalized to the maximum current recorded at the test V_m _of -20 mV. k = 4.6 ± 0.1 and n = 24 for WT; k = 4.5 ± 0.1 and n = 19 for A551T. C. V_m_dependence of steady-state intermediate and fast inactivation. For fast inactivation, k = 8.1 ± 0.2 and n = 9 for WT; k = 7.9 ± 0.2 and n = 8 for A551T. For intermediate inactivation, k = 16.2 ± 1.9 and n = 10 for WT; k = 13.9 ± 2.1 and n = 10 for A551T. Insets: V_m _protocols.

To examine whether the A551T mutation affected the V_m _dependence of channel activation, we next constructed the normalized conductance-V_m _relationship and found that the V_m_-dependent activation was the same in both the WT channel and the A551T mutant (Fig 3A). However, the V_0.5 _of the steady-state inactivation curve was shifted from -87.7 ± 0.6 mV in the WT to -92.7 ± 0.7 mV in the A551T mutant (p < 0.001) (Fig 3B), indicating enhanced inactivation in the mutant.

It has previously been shown that some SCN5A mutations associated with Brugada syndrome enhance the steady-state inactivation of Nav1.5 channels by promoting the entry of the channel into an intermediate inactivated state [9,19,20]. To determine whether the A551T mutant had a similar effect, we recorded the V_m _dependence of the intermediate inactivation state for the WT and A551T channels. Figure 3C shows that the fraction of channels entering the intermediate inactivation state was the same in the WT and A551T mutant at all V_m _tested. We then examined whether fast inactivation was affected by the A551T mutation and found that it significantly shifted the V_o.5 _by -6 mV (Fig. 3C, V_0.5 _= -78 ± 1.4 in WT; -84.2 ± 1.7 in A551T; p < 0.05). It has previously been shown that inactivation at a V_m _< -60 mV occurs primarily via transitions from the closed state [21]. Our results therefore suggest that the A551T mutation enhances entry into fast inactivation from the closed state.

**Figure 4:**
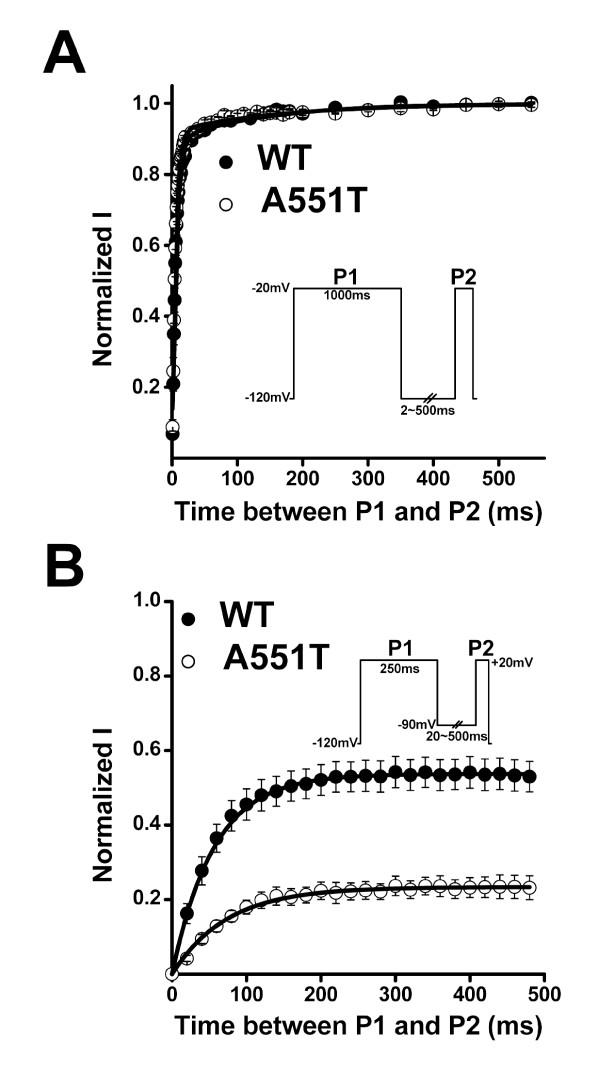
****Time-course of recovery from inactivation**. **A. Normalized currents were obtained by dividing the current obtained at the P2 pulse by that obtained at the P1 pulse with a holding and interpulse potential of -120 mV. B. Time-course of recovery recorded with a holding and interpulse potential of -90 mV. Insets: V_m _protocols (applied at 0.2 Hz). n = 7.

**Figure 5:**
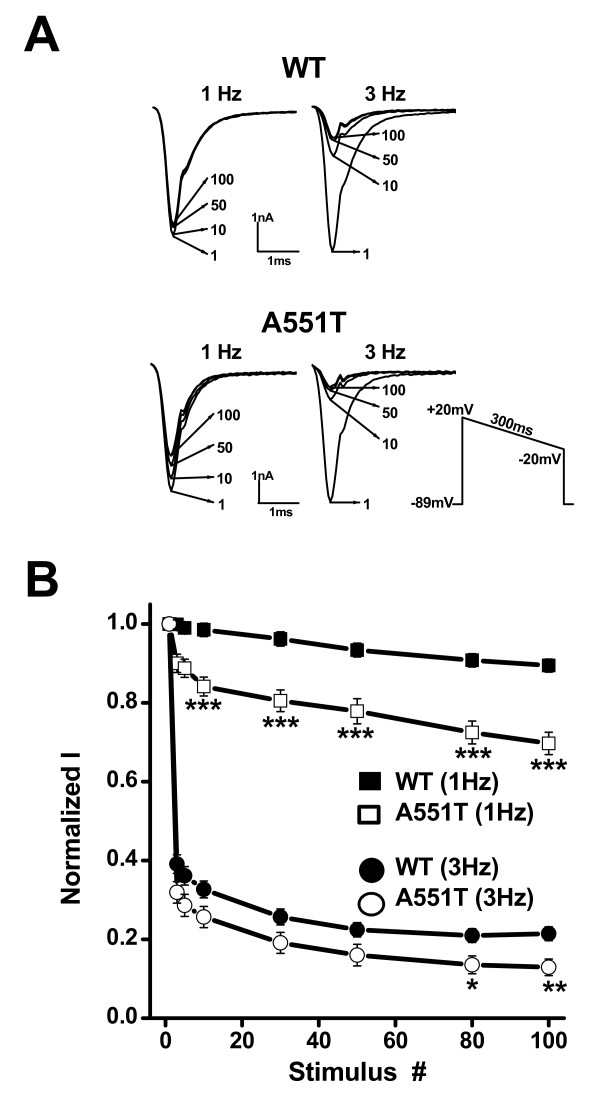
**Effects of repeated stimulation on Na^+ ^currents recorded from the wild-type and A551T mutant channels**. A. Current traces recorded using the ramp protocol (inset) mimicking the action potential during the 1st, 10th, 50th, and 100th depolarization at the indicated stimulation frequency. B. Normalized peak currents during a train of depolarizing pulses. The currents were normalized to that obtained at the first pulse. n = 11 – 17. **, p < 0.01; ***, p < 0.001.

Next, we studied whether recovery from inactivation was affected by the A551T mutation. Recovery was first evaluated at -120 mV after channels were activated and inactivated at -20 mV. Figure 4A shows that the time-course of recovery from inactivation were the same in the WT and the A551T mutant. However, when recovery was recorded at the physiological resting membrane potential, -90 mV, the time constant of the A551T mutant was 73.6 ± 4.6 ms, significantly larger than that of the WT (56.2 ± 4.4 ms, p < 0.05) (Fig. 4B). Furthermore, the percentage of channel recovery was 23 ± 1% for the A551T mutant, significantly lower than that for the WT (54 ± 2%, p < 0.001) (Fig. 4B). According to the data in Figure 3B, 38 ± 3% of WT and 62 ± 3% of A551T mutant channels entered the steady-state inactivation state at a holding potential of -90 mV. Together, the data show that 87% of WT and 61% of A551T channels recovered from the inactivation induced at +20 mV when recovery was examined at the physiological resting membrane potential. These results suggest that channel activity would decrease more in the A551T mutant than the WT with repeated stimulation at a constant frequency. In addition, due to the slower recovery rate in the A551T mutant than in the WT, Nav1.5 channel activity would be expected to decrease to a greater extent at rapid stimulation rates in the mutant than in the WT. To examine this hypothesis, we stimulated the WT and mutant at 1 and 3 Hz. Figure 5A shows the current traces recorded at the indicated frequency using a ramp protocol to mimic the depolarization duration of a cardiac action potential. As shown in Fig. 5B, when stimulated at 1 Hz, channel current amplitude was only slightly decreased (10 ± 2%) in the WT after 100 depolarizing ramp cycles, but was decreased by 30 ± 3% in the A551T mutant (p = 6.4 × 10^-6^, as compared to the value of the wild type), while the corresponding decreases at 3 Hz were 78 ± 1% and 87 ± 2% (p = 0.007, as compared to the value of the wild type).

Note that a depolarizing ramp instead of an action potential waveform was used to voltage clamp the cells in experiments described in Figure 5. Because the capacitance transient overlapped with the peak of Na^+ ^channels, it was difficult to quantitate the peak Na^+ ^current in action potential clamp. However, since our purpose was to examine the recovery of Na^+ ^channels from inactivation during the depolarization, the duration instead of shape of the repolarization is important. Therefore, using the depolarizing ramp protocol should not affect the conclusion.

### The effects of the A551T mutation are unlikely due to phosphorylation

**Figure 6:**
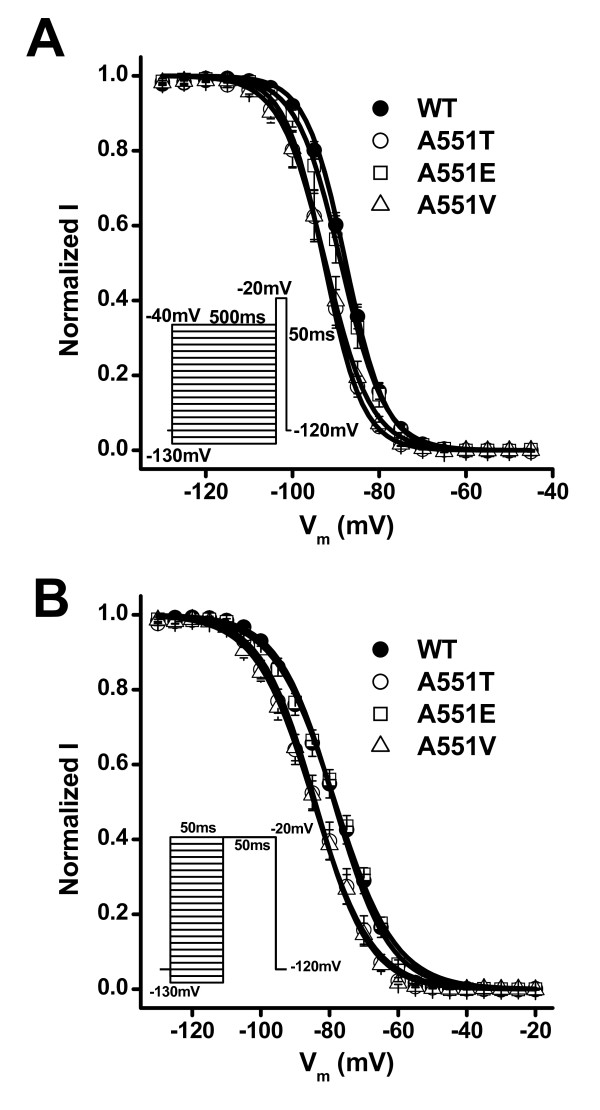
**Effects of the A551E and A551V mutations on the steady-state inactivation of Nav1.5 channels**. A. V_m _dependence of steady-state inactivation. k = 4.5 ± 0.1 and n = 19 for A551T; k = 5.2 ± 0.1 and n = 9 for A551V; k = 5.1 ± 0.1 and n = 8 for A551E. B. V_m _dependence of fast inactivation. k = 7.9 ± 0.2 and n = 8 for A551T; k = 8.5 ± 0.3 and n = 9 for A551V; k = 8.9 ± 0.4 and n = 8 for A551E. Insets: V_m _protocols (applied at 0.2 Hz).

As a first step to understanding the molecular mechanism underlying the effect of A551T on the NAV1.5 channel, we ran the kinase-specific eukaryotic protein phosphoylation site-predicting program on the NetPhos 2.0 server and found that A551T might be phosphorylated by casein kinase II (probability = 0.55). To examine whether phosphorylation is involved in the effects of the A551T mutation on the electrophysiological properties of Nav1.5 channels, we constructed the A551V and A551E mutants, the rationale being that valine is a similar size to threonine, but cannot be phosphorylated, whereas, if A551T is indeed phosphorylated and the negative charges of the phosphate are involved in the effects of the A551T mutant, the A551E mutant might have similar effects on Nav1.5 channels. Figure 6 shows that the A551 V mutant, like the A551T mutant, shifted the V_m _dependence of steady-state inactivation (V_0.5 _= -92.4 ± 0.2 mV, p < 0.001 as compared to that of wild type, Fig 6A) and fast inactivation (V_0.5 _= -84.6 ± 0.3 mV, p < 0.05 as compared to that of wild type, Fig. 6B) to the left, whereas the A551E mutant had no effect. These results strongly suggest that the effect of the A551T mutation on the left shift of the V_m _dependence of fast inactivation is not associated with phosphorylation at this site.

## Discussion

Mutations in the SCN5A gene can cause heart diseases, including Brugada syndrome, long QT syndrome, and isolated atrioventricular conduction block [1,3,4,9,22,23]. Brugada syndrome is characterized by an elevated ST-segment in the right precordial ECG leads and is associated with a high incidence of sudden death [6]. Previously, an A551T mutation in the Nav1.5 channel was identified in a proband with Brugada syndrome [16]. However, the functional alteration caused by the A551T mutation and its contribution to Brugada syndrome remain unknown. In this study, we compared the electrophysiological properties of the WT and A551T mutant coexpressed with the hβ_1 _subunit in HEK293T cells. We found that the current density of the A551T mutant was smaller than that of the WT. In addition, the A551T mutation increased the fraction of channels entering fast inactivation from the closed state at the resting membrane potential and decreased the fraction, and rate, of channel recovery at -90 mV from inactivation induced by depolarization. These changes in electrophysiological properties caused by the A551T mutation could potentially result in a significant decrease in the Na^+ ^current during phase 1 of the action potential and thus a change in the action potential dome in the epicardium, but not the endocardium, as the large transient outward current in the epicardium would be unopposed. This difference in changes in the dome would create a transmural voltage gradient that may be responsible for the ST-segment elevation associated with Brugada syndrome and might subsequently generate phase 2 reentrant extrasystole, predisposing the patient to ventricular tachycardia and ventricular fibrillation [24].

### Stimulation frequency effects

Brugada syndrome is characterized by a high incidence of sudden death in patients with structurally normal hearts [6]. Our results showed that Nav1.5 channel activities were dramatically reduced by the A551T mutation when stimulated continuously at 1 Hz (normal heart rate). The change in the action potential dome is critically dependent on the fine balance of currents, including I_Na_, I_to_, and I_Ca_, that are elicited during phase 1 of the action potential ^19^. It has been shown that the ST-elevation in Brugada syndrome is frequency-dependent. For example, a premature beat or faster pacing has been shown to restore the epicardial dome (due to slower recovery of I_to _from inactivation at higher stimulation rates) and thus decrease the ST-segment elevation [24]. In addition, the ST level decreases when the heart rate increases in patients with Brugada syndrome [25]. Thus, although we showed that the A551T mutation reduces Nav1.5 channel activity to a great extent at a greater stimulation rate, the effects of exercise on the patient remains unclear.

### Role of the DI-DII linker in fast inactivation

An important finding in this study is that an Nav1.5 mutation, A551T, in the cytoplasmic loop linking domains I and II increased fast inactivation from the closed state and reduced fractional, and rate, of channel recovery from inactivation, suggesting that the DI-DII linker may be involved in the fast inactivation gating process and its stability. The involvement of the DI-DII linker in regulating fast inactivation has been shown previously. An L567Q mutation of the Nav1.5 channel accelerates the inactivation kinetics of Nav1.5 currents and shifts the V_m _dependence of steady-state inactivation to the left [26]. In addition, a G514C mutant accelerates inactivation kinetics, but causes a positive shift in the V_m _dependence of steady-state inactivation [27]. Another mutant, L617F, induces a positive shift in the V_m _dependence of steady-state inactivation [28]. These previous studies and our present results provide evidence for a role of the cytoplasmic I-II linker in controlling the fast inactivation gate in Na^+ ^channels, a process that has been attributed to the binding of the fast inactivation particle in the DIII-DIV linker [29,30] to its docking sites in domains II-S4/S5 and IV-S4/S5 [31-34]. In this study, our results further suggest that the DI-DII linker may be involved in the regulation of the stability of the fast inactivation. It remains to be determined whether the DI-DII linker interacts directly with the fast inactivation particle or its docking sites, or allosterically regulates the inactivation gating process.

## Conclusion

Electrophysiological characterization of the Nav1.5 mutation, A551T, revealed that the cytoplasmic DI-DII linker regulates fast inactivation and its recovery. The decreased Nav1.5 activity caused by the A551T mutation supports the hypothesis that a reduction in Nav1.5 function is involved in the pathogenesis of Brugada syndrome. The structural-functional study of the Nav1.5 channel advances our understanding of its patho-physiolgocial function and provides potential preventive and therapeutic approaches to heart diseases.

## Competing interests

The authors declare that they have no competing interests.

## Authors' contributions

KCC conducted the experiments and analyzed the data, LPL conducted the experiments and wrote the manuscript, and RCS designed the experiments and wrote the manuscript.
